# Cone-Like Rectification Properties of cGMP-Gated Channels in Transmutated Retinal Photoreceptors of Nocturnal Geckoes

**DOI:** 10.1155/2014/942510

**Published:** 2014-11-23

**Authors:** Vittorio Vellani, Chiara Giacomoni

**Affiliations:** ^1^Dipartimento di Scienze Biomediche Metabolismo e Neuroscienze, Università di Modena e Reggio Emilia, via Campi 287, 41125 Modena, Italy; ^2^Dipartimento di Economia Scienze e Diritto, c/o Corso di Laurea in Ingegneria, Università della Repubblica di San Marino, Salita alla Rocca 44, 47890 Città di San Marino, San Marino

## Abstract

Photoreceptors of nocturnal geckoes are scotopic, with rod-shaped outer segments, and sensitivities to light similar to the one of retinal rods from other species of lower vertebrates. However, these cells are not rods, but they originated from cones of ancestral diurnal geckoes with pure-cone retinas, after being forced to adapt to a nocturnal behavior. Several interesting adaptations of these rod-like cones have been studied to date; molecular biology and functional studies confirmed that several proteins of the phototransductive cascade display structural and functional properties that indicate their origin from cones rather than from rods. In this paper, we investigate, with whole cell voltage clamp in the photoreceptor detached outer segment preparation, the voltage rectification properties of cGMP-gated channels in three species, *Gekko gecko*, *Tarentola mauritanica*, and *Hemidactylus frenatus*. We show that the current-voltage properties in the physiological voltage range are reminiscent of the ones of cGMP-gated channels from cones rather than from rods of other cold-blooded vertebrates. The origin and the relevance of the mechanisms investigated are discussed.

## 1. Introduction

The nature and the molecular origin of the differences between rod and cone function represent a highly relevant question in the study of phototransduction in vertebrates. Modern nocturnal geckos possess retinas with only rod-shaped photoreceptors; however, phylogenetic analysis of the* Gekkonidae* family suggests that both today's nocturnal and diurnal geckos evolved from a common ancestor with cone-only retinas. Therefore, photoreceptors of nocturnal geckos, despite the rod-like shape, ultrastructure, and function [1–3], are actually cones evolutionarily adapted to fulfill the role of rods and allow scotopic vision. This hypothesis was originally proposed by Walls and it is known as “transmutation theory” [4, 5].

Because of their intermediate physiological properties between rods and cones, photoreceptors from nocturnal geckos represent an outstanding model for the investigation of the physiological and molecular differences between these cell types, which may lead to general quantitative and qualitative cues on the mechanisms of phototransduction.

Qualitatively, exposure to light in both rods and cones causes a decrease in intracellular cGMP concentration ([cGMP]_i_), which in turn causes the closure of the cGMP-gated channel expressed in the plasma membrane of outer segment, the light-sensitive portion of vertebrate photoreceptors, and eventually produces hyperpolarization. The cGMP-gated channel is different in rods and cones [6], and the two different types of channels behave differently in several respects [7–9]. Previous investigation on gecko photoreceptors focused attention largely on the molecular structure of the visual pigments [10, 11] and more recently on the structure of other proteins of the phototransductive cascade [12]. No previous comparative study investigated the functional properties of the cGMP-gated channels in nocturnal gecko photoreceptors. In this study, we analyze the rectification properties of the light-sensitive current in the detached photoreceptor outer segment preparation (DOS) in three nocturnal gecko species,* Gekko gecko*,* Tarentola mauritanica,* and* Hemidactylus frenatus* and we show that current-voltage study displays cone-like features in the physiological voltage range.

## 2. Materials and Methods

### 2.1. Animals and Preparation

Geckos were obtained from Serpi&Co (Crespellano, Bologna, Italy), larval* Ambystoma tigrinum* from Charles D. Sullivan (Nashville, Tennessee, USA), and* Ambystoma mexicanum* from Schneider (Varese, Italy). Geckos were housed in cages and fed live crickets and mealworms. Salamanders were stored at 4°C in a cold room in oxygenated water and not fed. Animals were sacrificed after overnight dark adaptation by decapitation during anesthesia with urethane (1 g/kg i.p.). Using an infrared viewer, eyes were quickly removed and placed in ice-cold Ringer solution specific for amphibians or reptiles (see below). The caudal half of each eye was isolated with a razor blade, and retinas were peeled off the pigmented epithelium with fine forceps. Isolated rods and cones (*Ambystoma*) and detached outer segments (geckos) were obtained by gentle mechanical trituration using 2–6 passages through a fire-polished Pasteur pipette. Isolated photoreceptors retaining the inner segment could not be obtained in geckos using this method.

### 2.2. Solutions and Electrophysiology

Extracellular solution used for experiment on geckos contained 159 mM Na^+^, 3.3 mM K^+^, 1.0 mM Ca^2+^, 164.3 mM Cl^−^, 1.7 mM MgSO_4_, 10 mM glucose, and 2.8 mM Hepes, buffered to pH 7.4 with NaOH. For* Ambystoma* experiments, Ringer solution contained 120 mM Na^+^, 3 mM K^+^, 1.0 mM Ca^2+^, 1.5 mM Mg^2+^, 124 mM Cl^−^, 1.5 mM MgSO_4_, 10 mM glucose, 2.8 mM Hepes, and 5 mM TEACl buffered to pH 7.4 with NaOH.

Patch pipette solution contained 120 mM Asp, 130 mM K^+^, 6.05 mM Mg^2+^, 3 mM Na^+^, 1 mM GTP, and 5 mM ATP. Osmolarity of both extracellular and patch pipette (intracellular) solution was adjusted with sucrose at about 315–325 mOsm/Kg for gecko recordings and at about 240–250 mOsm/Kg for Ambystoma. The junction potential between pipette solution and external solution, carefully measured according to current methods described in Rispoli et al. [1], was about −9 mV and was corrected for in all recordings [13]. Patch clamp experiments were carried out with List EPC-7 amplifier (LIST medical instruments, Darmstadt, Germany) and Clampex software (Axon Instruments, Foster City, CA, USA). Patch pipettes were obtained from thick wall Blaubrand capillaries. Electrodes were pulled with a Sutter electrode puller model P-80 (Sutter Instruments, Novato, CA, USA) to a resistance of 2-3 MΩ. The patch clamp equipment was housed inside a light-tight Faraday cage in a dark room with dim red light [14]. In these conditions, it was possible to perform a full experiment without any light exposure to cells. The access resistance was typically 3 times the value of the open patch pipette. Recordings were discarded if access resistance was above 10 MΩ. About 60–80% of the access resistance was compensated by analog circuitry to minimize errors in voltage clamping. Cell capacitance ranged between 35 and 45 pF.

### 2.3. Isolation of the cGMP-Gated Component of the Light Sensitive Current

The whole cell current measured from gecko detached outer segments in the dark (dark current) was completely suppressed by exposure to saturating light delivered with an ultrabright green led (8.7*·*10^6^ h*ν* *·* *μ* ^−2^ sec^−1^, *λ* = 555 ± 5 nm). About 90–93% of the dark current was suppressed within 30–40 ms upon light saturation, while the remaining transient component of the current decayed exponentially to a steady level with a time constant of 0.2–1.2 s. The first component was considered the cGMP-gated component of the dark current [1] while the second component was due to the electrogenic activity of the 4Na:Ca,K exchanger extruding the residual calcium present in the detached outer segment. The sustained remaining current was leakage current and was subtracted. Input resistance was calculated from leakage current, and recording was discarded if value was below 1 GΩ.

## 3. Results

The current-voltage (*I*-*V*) relationship of the cGMP conductance in amphibian rods displays large outward rectification [15, 16], very well fitted by an exponential function with a voltage constant between +10 and +15 mV [15]. The *I*-*V* curve in cones shows largely similar behaviour for depolarizing voltages, but it also shows inward rectification [17, 18]. The *I*-*V* curve in both rods and cones is the one expected if the rate limiting step of the cGMP channel permeation was the passage through a single energy barrier located at a fraction *γ* of the transmembrane voltage field and if cations permeated the channel in divalent form, or in pairs [15, 19]. It is well agreed that this behaviour is due to the partial block of the channel by physiological divalent cations, Ca^2+^ and Mg^2+^. In fact, the rectification behaviour depends on the presence of divalent cations on either side of the membrane. Their removal causes the disappearance of rectification, and the *I*-*V* curve becomes linear [20, 21].

The *I*-*V* relationship of the cGMP-gated current in both rods and cones can be interpolated by the following equation [18]:

(1) IV=exp⁡1−γV−VrevV0 −exp⁡−γV−VrevV0,

where *I* is normalised transmembrane current, *V* is membrane potential, *γ* is the symmetry constant mentioned above, *V*_rev_ is reversal potential, and *V*_0_ is the voltage constant [19]. The value of *γ* in amphibian photoreceptors is about 0 in rods and 0.35 in cones [18]; *V*_0_ ranges between +10 and +15 mV in both cell types [18].

The *I*-*V* relationship of the cGMP-gated conductance was studied in the detached photoreceptor outer segment preparation (DOS) with the whole cell configuration of the patch clamp technique [1]. In this preparation, DOSs are obtained by gentle mechanical trituration of dark-adapted retinas with no alteration of membrane channel proteins due to proteolytic enzymes. DOS, even if kept in the dark, cannot produce enough ATP and GTP to support phototransduction, and therefore the light-sensitive current is absent. However, when recorded in whole cell with the patch clamp technique, a significant diffusional exchange between patch pipette solution and cytoplasm takes place, and large amounts of ATP and GTP can be washed-in from the patch pipette solution. In these conditions, if maintained in complete darkness, the light-sensitive current (carried mainly by Na^+^ and Ca^2+^ ions) recovers completely reaching stability within a couple of minutes [1]. During whole cell recording, the homeostasis of DOS intracellular Ca^2+^ is controlled by the Na^+^:Ca^2+^,K^+^ exchanger as in intact photoreceptors [22, 23], the inflow of Na^+^ is counterbalanced by the wash-out via the patch pipette, which in this way replaces the activity of the Na^+^/K^+^ pump normally present in the inner segment but not in the outer segment of vertebrate photoreceptors. The DOS recorded in this way therefore is an excellent preparation to isolate and investigate the light sensitive, cGMP-gated current expressed in photoreceptors, as no other significant conductance is present in DOS [22].

The DOSs from* Gekko gecko *(*Gg*),* Tarentola mauritanica* (*Tm*) and* Hemidactylus frenatus* (*Hf*) used in our recordings were all rod-shaped, of size similar to the one of rods from other lower vertebrates [24]. All recordings were obtained from single, isolated DOS, while twin DOSs were avoided. Similar recordings were performed in rods and cones from the tiger salamander (*Ambystoma tigrinum*) and from axolotl (*Ambystoma mexicanum*) in order to check the *I*-*V* behaviour of the cGMP-gated current, which was comparable to the one reported in the literature (not shown).

**Figure 1:**
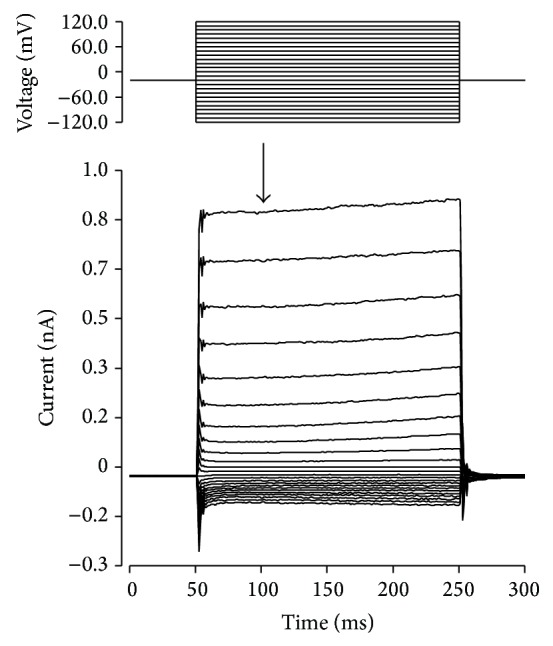
cGMP-gated current amplitude during voltage step protocol. Typical whole cell voltage clamp recording of a gecko photoreceptor detached outer segment (*Tarentola mauritanica*) performed in the dark. Upon exposure to bright light, most current disappeared and only a negligible amount of leakage current remained. Arrow indicates the time (50 ms after voltage change) when current amplitude was measured to obtain the *I*-*V* plot, before any time-dependent change of cGMP-gated current occurred. Further details in text.

After obtaining the whole cell recording from a DOS in complete darkness, the dark current was allowed to develop and stabilize for 5 minutes, and then a voltage step protocol was applied (Figure 1). Steps had a duration of 200 ms and were applied at 2 s intervals. Current amplitude during this protocol did not change if steps were applied at 5 or 10 s intervals. Current amplitude at different voltages was highly repeatable, and the same depolarizing step induced basically identical current in all trials (not shown). At the end of the experiment, each DOS was exposed to saturating light, which totally blocked the cGMP-gated current. cGMP-gated current was suppressed within 30–40 ms. After that, the transient component of the dark current produced by Na:Ca.K exchange activity and the leakage current was subtracted (see Section 2). In these conditions, we confirmed that no other significant conductance was present in each individual DOS [1, 22]. Input resistance (*R*_in_) at the end of the experiment was then calculated by applying again the voltage step protocol. Only recordings that showed Rin > 1 GΩ were considered for analysis.

**Figure 2:**
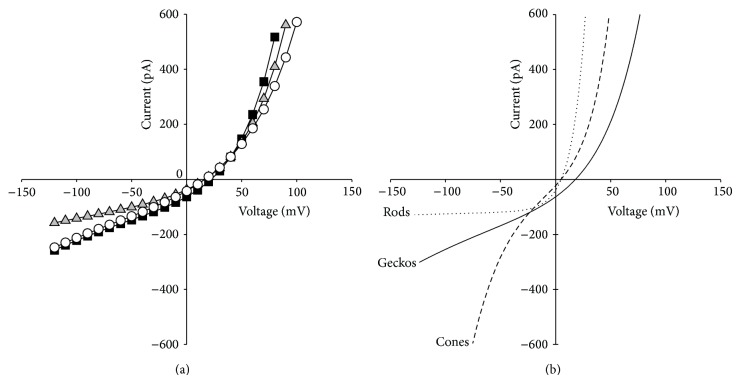
*I*-*V* relationship of light-sensitive (cGMP-gated) current recorded from gecko photoreceptor DOS. (a) *I*-*V* relationship of cGMP-gated current in a single, representative DOS from the nocturnal geckos* Gekko gecko* (black squares),* Tarentola mauritanica* (white circles), and* Hemidactylus frenatus* (grey triangles). Each curve shows a single trial from a single DOS. Data were fitted with (1) (continuous lines). Average parameters obtained are in Table 1. (b) Numerical simulation according to (1) of the *I*-*V* behavior of the cGMP-gated current in amphibian rods (dotted line), in catfish cones (dashed line), and in nocturnal geckos (solid line).

Figure 2 shows the *I*-*V* behaviour of cGMP-gated current amplitude in recordings from DOS isolated from retinas of* Gg*,* Tm,* and* Hf*. Equation (1) was fitted to data obtained in each experiment, and the values of the parameters were averaged. Results are summarised in Table 1 (mean ± SD) and compared to results in rods and cones from lower vertebrates from the literature.

While in rods rectification occurs only in the outward directions, in cones there is a clear inward component, as shown in Figure 2(b) [18]. The cGMP-gated channels of gecko photoreceptors display a clear outward rectification, but a smaller inward rectification, due to the smaller value of the *γ* parameter of (1) compared to cones. The overall behavior therefore, in some aspects, is somehow more similar to the one of cones rather than that of rods in the physiological voltage range (see Figure 2(b)), and in other aspects is unique, different from both rods and cones. In fact (see Table 1), the outward rectification parameter *V*_0_ has got higher values in geckos compared to the one in cones, and the reversal potential of the cGMP-gated current in nocturnal geckos is higher than the one reported in rods and cones from fish, amphibians, and lizards [17, 18, 25, 26].

## 4. Discussion

Rods are scotopic photoreceptors, with sensitivity pushed to the single photon level, the physical limit of detection. Cones instead are photopic receptors, with several redundant mechanisms which evolved to adapt phototransduction gain to bright light and avoid saturation. The inward rectification properties of the cGMP-gated channels expressed in cones represent one of these mechanisms promoting adaptation to light. In cones in fact, the inward rectification of the light-sensitive current occurs in the physiological voltage range. During hyperpolarization in nonsaturating light, the partial current suppression caused by intracellular cGMP reduction will be counteracted by the increase in the residual cGMP-gated current caused by its inward rectification properties, which will ultimately reduce the voltage response to light of the cone cells. We showed that a similar (see the *γ* parameter in Table 1), although, less evident inward rectification property is also present in photoreceptors in nocturnal geckos. In the inward direction in the voltage range investigated, the cGMP-gated current displays an apparently ohmic behaviour, which in fact is different from the one of rods of other lower vertebrates (Figure 2(b)), and is reminiscent of their origin from ancestral cones. It is interesting that although nocturnal geckos are active mainly at night, they can see perfectly and interact with the environment in bright daylight, which indicates that although all of their photoreceptors are rod-like in morphology, and very sensitive to dim light, they can adapt to light extremely effectively [27, 28]. The presence of a remaining component of the inward rectification present in cones in the light-sensitive current of nocturnal gecko photoreceptors contributes to their light-adaptation capacities and also provides a novel, functional piece of evidence in support of Walls' transmutation theory [4].

The outward rectification of the light-sensitive current in nocturnal geckos is significantly less steep in comparison to the one reported in the literature in rods and cones [18, 20]. This indicates that the energy barrier that monovalent cations need to overcome in the ion channel pore to counteract block by divalent cations is lower in comparison to rods and cones.

The reversal potential of the light-sensitive current in nocturnal gecko photoreceptors is larger than the one reported in the literature in rods and somewhat larger even than the one reported for cones from other lower vertebrates [15, 16, 18]. This may indicate a higher permeability of the gecko cGMP-gated channel to calcium compared to other lower vertebrates, which indeed has been observed in preliminary experiments [1, 15, 24, 29] rather than a higher permeability to K^+^ in comparison to Na^+^ [21]. It is possible though that the higher reversal potential in geckos may be explained by hindered intracellular diffusion of Na and K^+^ to/from the patch pipette during DOS recording. Na and K gradients in fact are used as a source of free energy by the Na:Ca,K exchanger, and the intracellular stack of disks in DOSs may significantly slow down diffusion [22].

In conclusion, in this paper, we provide a novel, functional evidence of the presence of mechanisms in rod-shaped photoreceptors from nocturnal geckos that are reminiscent of their origin from ancestral cones. The molecular analysis of the retinal cGMP gated channel recently performed in* Gekko gecko* identified cone-like structural features [12], which agree with our observations.

## Figures and Tables

**Table 1:** Parameters describing the rectification properties of the cGMP-gated channel in nocturnal gecko photoreceptor DOS and in rods and cones.

Cell type	*γ*	*V*_rev_ (mV)	*V*_0_ (mV)
*Gg* DOS (*n* = 12)	0.217 ± 0.013	16.6 ± 0.9	30.3 ± 0.4
*Tm* DOS (*n* = 18)	0.195 ± 0.008	18.8 ± 1.9	29.6 ± 0.6
*Hf* DOS (*n* = 12)	0.183 ± 0.006	17.1 ± 1.0	29.8 ± 0.4
Cones [18]	0.35	13.5 ± 2.9	12.5
Rods [18]	0.01	0–10	12.5–25
